# L-Type Ca^2+^ Channel Function Is Linked to Dystrophin Expression in Mammalian Muscle

**DOI:** 10.1371/journal.pone.0001762

**Published:** 2008-03-12

**Authors:** Oliver Friedrich, Frederic von Wegner, Jeffrey S. Chamberlain, Rainer H. A. Fink, Petra Rohrbach

**Affiliations:** 1 Medical Biophysics, Department of Systems Physiology, Institute of Physiology and Pathophysiology, University of Heidelberg, Heidelberg, Germany; 2 Department of Neurology, University of Washington, Seattle, Washington, United States of America; 3 Department of Parasitology, Hygiene Institute, University of Heidelberg, Heidelberg, Germany; Vrije Universiteit Amsterdam, Netherlands

## Abstract

**Background:**

In dystrophic mdx skeletal muscle, aberrant Ca^2+^ homeostasis and fibre degeneration are found. The absence of dystrophin in models of Duchenne muscular dystrophy (DMD) has been connected to altered ion channel properties e.g. impaired L-type Ca^2+^ currents. In regenerating mdx muscle, ‘revertant’ fibres restore dystrophin expression. Their functionality involving DHPR-Ca^2+^-channels is elusive.

**Methods and Results:**

We developed a novel ‘in-situ’ confocal immuno-fluorescence and imaging technique that allows, for the first time, quantitative subcellular dystrophin-DHPR colocalization in individual, non-fixed, muscle fibres. Tubular DHPR signals alternated with second harmonic generation signals originating from myosin. Dystrophin-DHPR colocalization was substantial in wt fibres, but diminished in most mdx fibres. Mini-dystrophin (MinD) expressing fibres successfully restored colocalization. Interestingly, in some aged mdx fibres, colocalization was similar to wt fibres. Most mdx fibres showed very weak membrane dystrophin staining and were classified ‘mdx-like’. Some mdx fibres, however, had strong ‘wt-like’ dystrophin signals and were identified as ‘revertants’. Split mdx fibres were mostly ‘mdx-like’ and are not generally ‘revertants’. Correlations between membrane dystrophin and DHPR colocalization suggest a restored putative link in ‘revertants’. Using the two-micro-electrode-voltage clamp technique, Ca^2+^-current amplitudes (i_max_) showed very similar behaviours: reduced amplitudes in most aged mdx fibres (as seen exclusively in young mdx mice) and a few mdx fibres, most likely ‘revertants’, with amplitudes similar to wt or MinD fibres. Ca^2+^ current activation curves were similar in ‘wt-like’ and ‘mdx-like’ aged mdx fibres and are not the cause for the differences in current amplitudes. i_max_ amplitudes were fully restored in MinD fibres.

**Conclusions:**

We present evidence for a direct/indirect DHPR-dystrophin interaction present in wt, MinD and ‘revertant’ mdx fibres but absent in remaining mdx fibres. Our imaging technique reliably detects single isolated ‘revertant’ fibres that could be used for subsequent physiological experiments to study mechanisms and therapy concepts in DMD.

## Introduction

Duchenne muscular dystrophy (DMD) is a common X-chromosomal hereditary disease that involves progressive muscle wasting and eventually results in immobility and death from respiratory and cardiac failure early in adulthood [1], [2]. Mutations that involve premature stop-codons or shifted reading frames of the ∼2.5 Mb-long dystrophin gene are primarily responsible for the complete absence of the 427 kDa cytoskeleton protein dystrophin in DMD [3]-[5]. Although dystrophin was found to be a major mechanical linkage protein between the extracellular matrix and the intracellular cytoskeleton [3], [6], [7], its implications for the pathophysiological mechanism have been more complex than originally anticipated. On the one hand, dystrophin has been shown to stabilize the sarcolemma against stress-induced muscle damage [8], [9]. In its absence, increased membrane damage triggers repetitive cycles of degeneration and regeneration. Incomplete regeneration typically results in an abnormal morphology of dystrophic skeletal muscle (i.e. branching and splitting [10]). On the other hand, there have been numerous reports that suggest dystrophin may regulate other cellular targets [11], e.g. ec-coupling and Ca^2+^ homeostasis (e.g. [12]–[16]), mitochondrial function [17], motor protein interaction [18], [19] or gene transcription 20, 21. From these studies, dystrophin has been implicated in the regulation of cellular signalling cascades either directly by regulating membrane-associated proteins, including ion channels [13], or indirectly via second messenger cascades [22], [23]. For example, lack of dystrophin has been shown to cause aberrant mechanotransduction [24]. Furthermore, cytosolic Ca^2+^ homeostasis is impaired by alterations of ion channels and pumps that may affect intracellular Ca^2+^ concentration [12]–[15], [25]–[27]. However, from the controversy concerning different Ca^2+^ entry pathways and how they might affect intracellular Ca^2+^ levels [28], [29], it has become apparent that not only different experimental conditions (e.g. [30], [31]), but also the developmental stage and the age of the muscle preparation are crucial determinants of ion channel function [32], [33]. In the mdx mouse, the most frequently used animal model for DMD that contains a nonsense point mutation in exon 23, the age dependence of the muscle proteome was recently quantified [34].

In wild-type skeletal muscle, L-type Ca^2+^ channels (DHPR, Dihydropyridine receptors) in the transverse-tubular membrane may contribute to Ca^2+^ influx during prolonged muscle activation (i.e. tetanic stimulation, [35], [36]) or store depletion [37], although under normal conditions of single twitches they serve as voltage sensors to induce Ca^2+^ release from the sarcoplasmic reticulum rather than acting as conducting ion channels [38]. Nevertheless, the properties of the L-type Ca^2+^ channel well reflect the muscle fibre's physiological state that might also be altered in other pathophysiological conditions [39], [40].

We have recently started to investigate L-type Ca^2+^ channel properties in single skeletal muscle fibres from mdx mice over an age range from two to 18 months [25]. Our results were interpreted in terms of a model in which dystrophin may influence L-type Ca^2+^ channels properties. We also hypothesized that some of the changes we found in L-type channel properties in mdx fibres with age might be correlated with the presence of ‘revertant’ fibres in aged mdx mice that show spontaneous mutations that restore the expression of dystrophin [41].

As the mdx mouse has a defined genetic background for the dystrophic phenotype, it is a very suitable model not only to study pathophysiological mechanisms of DMD but also to functionally establish the benefits of gene therapy strategies [3]. One approach is to design expression cassettes of exons from the dystrophin gene that result in the expression of shorter, mini- or even micro-dystrophins [3]. Such smaller dystrophins can be stably expressed in transgenic strains [42] or be used for vector-mediated transfection [43]. In our previous study, we could show that mini-dystrophin can fully restore the decreased L-type Ca^2+^ currents seen in mdx fibres, regardless of age.

In the present study, we followed our working hypothesis of L-type Ca^2+^ channel (DHPR)–dystrophin interaction. Our recent results combined with data on cardiac muscle [44], [45] encouraged us to further characterize the influence of dystrophin on Ca^2+^ channels. We were particularly interested whether, similar as for cardiac muscle [44], a colocalization of both protein complexes was present in skeletal muscle. For this purpose, we developed a new ‘in situ’ confocal immuno-fluorescence imaging technique in unfixed single adult muscle fibres. Our results in permeabilized skeletal muscle fibres from wild-type and mini-dystrophin expressing animals show that dystrophin significantly colocalizes with DHPRs in the triad region. In the mdx phenotype, however, colocalization is diminished in most, but not all, fibres. Quantification of the membrane-bound dystrophin signal patterns in single fibres from middle-aged and older mdx mice revealed that some of these latter mdx fibres had significantly increased membrane dystrophin signals that were similar to those in wt fibres (‘wt-like’) and were thus identified as ‘revertants’. When evaluating the distribution of maximum peak L-type Ca^2+^ current amplitudes (i_max_) in mdx mice, some fibres from aged mdx mice clustered at amplitudes that are more ‘wt-like’ while the remaining fibres showed the small i_max_ amplitudes that are exclusively seen in fibres from young mdx mice. These novel findings suggest that the ‘revertant’ fibres expressed in aged mdx skeletal muscle cells may also be able to restore DHPR function.

## Methods

### Genetic strains and preparations

The genetic backgrounds of single fibres used here are described in [25]. C57/SV129 wild-type (wt) mice served as a control. mdx mice represent an animal model for DMD [46], and MinD mice are a transgenic strain with the mdx background (CVBA3', [42]) expressing the Δexon17–48 mouse mini-dystrophin construct with a molecular weight of 228 kDa [42], [47]. Mdx mice of both sexes were used. Females were homozygous for the mdx genotype. Animals were sacrificed by exposure to a 5% CO_2_ atmosphere. All experiments were performed according to the guidelines laid down by the local Animal Care Committee of the University of Heidelberg. Single isolated fast-twitch fibres from interossei muscles were obtained after collagenase treatment (1.5 mg/ml, 30 min at 30°C, collagenase type IA, Sigma-Aldrich, Taufkirchen, Germany) as previously described [48]. All subsequent experiments were conducted at room temperature (21–23°C).

### Voltage clamp experiments and data analysis

Two-micro-electrode voltage clamp experiments in a total of n = 287 single fibres under hypertonic extracellular conditions in wt, mdx and MinD fibres of matching age groups were performed. The conditions have the advantage of minimised tubular Ca^2+^ depletion due to swelling of the t-tubules that might otherwise affect current kinetics and amplitudes [49], [50]. The hypertonic conditions do not induce artefacts to the L-type Ca^2+^ currents (i_Ca_) as previously shown in the three genotypes [25]. In our previous work, we focussed on I-V relationships, voltage-dependent inactivation and deactivation. Here, we investigated the age dependent maximum peak current distributions i_max_ from all our single fibre data (this and our previous study). All raw data from electrophysiology and immuno-fluorescence microscopy were collected in scatter plots to investigate data distributions. Student's t-test was used for normal distributions. P<0.05 was considered significant. In case of no normal distribution, data are described with the robust parameters median and quartiles. i_Ca_ data were not normalized to capacitance as capacitance measurements were only available in some fibres. However, we have shown previously that capacitance data are similar in the three strains at different ages [25] and should not affect our conclusions. As steady-state activation of L-type Ca^2+^ channels at a given membrane potential, d_∞_, is a determinant of i_max_ amplitudes [51], a possible shift in activation curves might explain amplitude differences between strains. d_∞_ was determined from i_Ca_-V-plots of each individual fibre, as described in [48]:
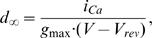
(eq.1)with the maximum conductance g_max_ derived from a linear fit to the i_Ca_-V-plot at larger potentials, the interception of which with the abscissa also yields the reversal potential V_rev_. A Boltzmann-fit was drawn through the d_∞_-curve and the half-activation potential d_0.5_ and the slope factor k was extracted. For each strain and age bin, the mean activation curve was then reconstructed from the mean values of their d_0.5_ and k parameters. This perfectly described the mean d_∞_ data for each bin (see Results).

### Solutions

Normal saline for the preparation of muscles and isolation of single fibres contained (mM): NaCl 140, KCl 4, CaCl_2_ 2, MgCl_2_ 1, Hepes 10, pH 7.40. Hypertonic 10 mM Ca^2+^ containing solution for recording of i_Ca_ contained: TEA-Br 146, Ca-acetate 10, KCl 1, Mg-acetate 1, CsBr 5, 4-aminopyridine 5, 3,4-di-aminopyridine 5, Hepes 10 and sucrose 300, pH 7.4, osmolarity 645 mosmol/l. Mammalian skeletal muscle relaxing solution contained: K-glutamate 125, MgCl_2_ 6, CaCl_2_ 0.13, Na_2_ATP 5, Na_2_CP 10, EGTA 1, Hepes 10, glucose 10, pH 7.0. All compounds were purchased from Sigma Aldrich (Taufkirchen, Germany) except where otherwise stated. To permeabilize the sarcolemma of intact fibres, saponin (Fluka Chem., Buchs, Germany) was added to a final concentration of 0.01% (w/v) to the relaxing solution.

### Antibodies and immunological staining

Immuno-colocalization experiments were performed using monoclonal mouse or rabbit primary antibodies. Mouse anti-dystrophin antibodies against the intracellular rod-domain (NCL-DYS1) and the C-terminal domain of dystrophin (NCL-DYS2) were from Novocastra Laboratories (Newcastle upon Tyne, UK). Lyophilized primary anti-calcium channel antibody (Pan α1 subunit), raised in rabbit and recognizing all types of α1 subunits of voltage gated calcium channels (amino acids 1382-1400) of rat and mouse skeletal muscle (Dihydropyridine-receptors, DHPR) was supplied from Sigma Aldrich (Steinheim, Germany). ALEXA-Fluor 633 goat anti-mouse IgG (H+L, 2 mg/ml) and BODIPY FL goat anti-rabbit IgG (H+L, 1 mg/ml) were used as secondary antibodies (MoBiTec, Göttingen, Germany). For staining, primary antibodies were diluted 1:25 (anti-DYS1/2) or 1:100 (anti-DHPR) and secondary antibodies 1:100 in relaxing solution that contained 1% bovine serum albumin (BSA, Sigma Chemicals) to saturate non-specific binding sites.

After the transfer of approx. 20–30 single fibres to the recording chamber, the normal saline was slowly exchanged to relaxing solution, followed by the addition of saponin for 2 min to chemically permeabilize the sarcolemma. Saponin permeabilizes cholesterol rich membranes by formation of membrane pores [52]. Mammalian skeletal muscle fibres are known to preserve an intact contractile apparatus and intracellular Ca^2+^ regulation under these conditions [53]. A putative protein-protein link between dystrophin and DHPR should, therefore, not be affected by the treatment.

After washout of the detergent, the preparation was incubated with the primary antibodies for 105 min at room temperature, washed five times and the secondary antibodies applied. After 30 min incubation at room temperature, the excess secondary antibodies were washed out by rinsing with fresh relaxing solution/1% BSA. After the successive washing procedures, approximately 50% of fibres were lost or damaged. Immuno-fluorescence microscopy was performed in the remaining permeabilized, i.e. non-fixed, cells. Non-specific binding of the fluorescently labelled antibodies was quantified by imaging several single fibres after incubation of the secondary antibodies alone. These negative controls were used to correct all immuno-fluorescence images for the average non-specific antibody signals (see below).

### Confocal immuno-fluorescence microscopy of dystrophin and DHPR and multiphoton second harmonic imaging of myosin

The immuno-labelled samples were mounted on the stage of an inverted Leica SP2 confocal microscope (Leica Microsystems, Mannheim, Germany) equipped with an Ar^+^ ion laser (488 nm excitation) and a HeNe laser (633 nm excitation). BODIPY labelled DHPR and ALEXA labelled dystrophin were excited at 488 nm and 633 nm and fluorescence collected from 510–570 nm and 645–700 nm, respectively. Laser power of the Ar^+^ and the HeNe laser as well as the PMT settings were kept constant throughout for each fibre. Between individual fibres, laser power was kept constant to avoid photodamage to the fibres during series of experiments. PMT gain was individually adjusted (∼10% changes in gain) for both channels simultaneously. This small difference in PMT gains, however, does not affect our image analysis, as signals were corrected for background and both channels were ratioed for the DHPR and dystrophin colocalization values (see below). This procedure removes noise arising from differences in PMT gain of both channels. Crosstalk between the channels was excluded by alternately switching off the lasers and collecting fluorescence in both wavelength bins (not shown). Image analysis was carried out using three images per fibre, obtained at different magnifications in order to exclude the zoom factor as a confounding variable. At the lowest magnification factor (overview images), images were recorded with 512×512 pixels/image and higher magnifications at 1024×1024 pixels/image resolution. Individual images were online-averaged (4–12 consecutive images) during image acquisition to increase the signal-to-noise ratio. In a few single fibres, myosin was additionally visualized with *second harmonic generation* (SHG) imaging [54] using a ps Ti:Sa laser (Tsunami, Spectra Physics, Mountain View, CA, USA) at 880 nm. The forward scattered SHG signal was separated from the simultaneously excited BODIPY signal with a band-pass filter (450 nm).

Combination of both techniques (i.e. voltage-clamp and immuno-fluorescence) in the same individual muscle fibre is currently not possible. Fibres permeabilized for immunostaining cannot be used for subsequent electrophysiological measurements that require an intact sarcolemma. On the other hand, the permeabilization and confocal imaging procedure requires structurally intact fibres. After a complete stimulation protocol, involving maintained depolarisations, single fibres do not tolerate a subsequent permeabilization and staining protocol. Furthermore, removal of the microelectrodes produces significant membrane damage, leaving the cytoskeletal-membrane architecture in an undefined state. Thus, voltage-clamp and imaging data, as presented in this work, are obtained from different fibres and statistically evaluated.

### Image analysis

#### Signal correction for non-specific antibody binding from negative controls

Non-specific binding of secondary antibodies was quantified in ten wt single fibres at up to three magnifications each. Representative extracellular and intracellular regions-of-interest (ROIs, >2000 pixels each) were manually selected in each image and their mean intensity was extracted from the fluorescence intensity histograms (ImageJ software, NIH, http://rsb.info.nih.gov/ij/). The mean intensity ratio ρ_u_ from the intracellular to the extracellular region was calculated and averaged over all images (*ρ̅*_u_). This represents the overall non-specific binding of fluorescently labelled secondary antibodies. Individual images from the complete labelling protocol, i.e. involving primary and secondary antibodies, were corrected by subtracting *ρ̅*_u_ from their corresponding ratios ρ. All following image procedures are performed on the corrected images.

#### Colocalization of DHPR and dystrophin (Dys) signal intensities

The threshold of both DHPR and Dys fluorescence channels was determined from the background region lying outside the fibres using the statistics of those regions' intensity histograms. A statistical cut-off was set to mean+2 SD, where SD denotes the standard deviation. Thresholded images were binarized and multiplied to obtain the pixels that were significantly colocalized in both channels. Their pixel count was divided by the pixel count in the binarized DHPR channel as reference to yield the percentage of DHPR colocalized with respect to dystrophin.

#### Quantification of membrane dystrophin signal intensities

In wt and MinD fibres, a large but variable fraction of the dystrophin (Dys) signal originates from beneath the plasma membrane. To quantify relative dystrophin signal intensities that originate from membrane and intracellular regions, the following imaging procedure was applied: for each fibre, three membrane regions (*M_1_, M_2_, M_3_*), an intra- (*R_In_*) and an extracellular region of interest (*R_Out_*) were manually selected and their respective mean intensities calculated. Subsequently, the four mean intensity ratios *M_1_/R_out_, M_2_/R_out_, M_3_/R_out_ and R_In_/R_Out_* were corrected for non-specific binding of secondary antibodies (see above). Finally, the three corrected (*M_1..3_/R_Out_)* ratios were averaged (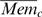) and the difference 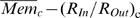 was used to quantify the membrane density of dystrophin over the internal density. This approach allowed elimination of possible effects from poor overall staining in some fibres over well-stained fibres. We chose to select three membrane regions to account for heterogeneous (‘patchy’) Dys staining observed in some mdx fibres (see Results).

## Results

### Tubular DHPR signals alternate with A-band SHG signals that originate from myosin

**Figure 1:**
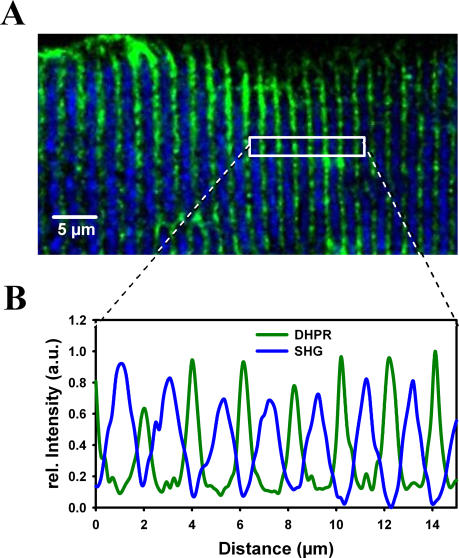
Alternating pattern of DHPR immuno-fluorescence signals and *Second Harmonic Generation* signals using multiphoton excitation in unfixed single wt fibres. A, overlay of DHPR immuno-fluorescence (green) and the SHG signal (blue) simultaneously recorded in a representative wt fibre excited with a pulsed Ti:Sa laser at 880 nm. The signal intensity profile plot (B) confirms the tubular origin of the DHPR signal that alternates with the SHG-signal arising from the myosin within the A-bands.

T-tubules are located adjacent to the myosin filaments in mammalian skeletal muscle. To verify the correct tubular localization of the DHPR signal, both the DHPR signal and the SHG signal arising from myosin were obtained with 2-photon excitation at 880 nm. Fig. 1 A shows such an overlay image of the DHPR (green) and the SHG (blue) channel from a representative wt single interosseus muscle fibre. The signal intensity profiles from both channels within the ROIs shows alternating signal intensities within the sarcomere. This confirms the correct (t-tubular) origin of the DHPR fluorescence and the specificity of the antibodies used. Similar results were obtained in several other fibres tested.

### DHPR and dystrophin colocalization in wt, mdx and MinD fibres

**Figure 2:**
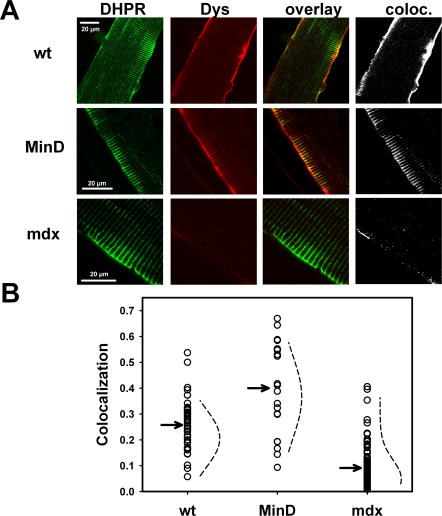
Colocalization of DHPR and dystrophin signals in single wt, MinD and mdx fibres. A, representative examples of ‘in-situ’ DHPR (green) and dystrophin (Dys, red) immuno-fluorescence signals in single 10 months old wt, MinD and mdx fibres. The overlay of both DHPR and Dys signals and the colocalization images are also shown. Colocalization is substantially reduced in mdx fibres. B, Colocalization values from 20 to 75 images of each strain show smaller median values (arrows) for mdx fibres compared to wt fibres and MinD fibres. Note that a few mdx fibres show colocalization values comparable to wt fibres. The dotted lines are Gaussian fits to the data indicating that they are normally distributed in the strains.

Representative images of a single interosseus fibre from a wt, MinD and mdx mouse (age: 10 months) are shown in Fig. 2 A. DHPR and dystrophin immuno-fluorescence, the overlay and the calculated colocalization images are shown. Images were corrected for non-specific binding of secondary antibodies, as detailed in Methods. There is a strong dystrophin signal originating from the membrane area in both wt and MinD fibres but almost no signal is found in the mdx fibre. One should note that dystrophin signals also extend to within the fibre, i.e. the triad regions (colocalization image). The mean relative colocalization of DHPR with respect to dystrophin was 0.27, 0.52 and 0.05 for the wt, MinD and mdx fibre displayed. Note that this ratio corresponds to colocalized dystrophin-DHPR complexes within single fibres rather than the percentage of dystrophin-positive fibres. The trend of very low colocalization ratios in mdx fibres and restored values in MinD fibres was confirmed (Fig. 2 B) in 44 images from n = 13 wt (0.26±0.09, mean±SD), 21 images from n = 7 MinD (0.4±0.17) and 74 images from n = 31 mdx fibres (0.10±0.07). Data were normally distributed in the three strains (dotted lines in Fig. 2 B). Colocalization ratios were independent of the zoom-factor used in the different images (data not shown). It should be noted that the diminished colocalization values in mdx fibres represent a robust quality control for our imaging technique, as the marked reduction in colocalization of DHPR with respect to dystrophin is expected due to the absence of dystrophin.

### Ca^2+^-current distributions in mdx mice

**Figure 3:**
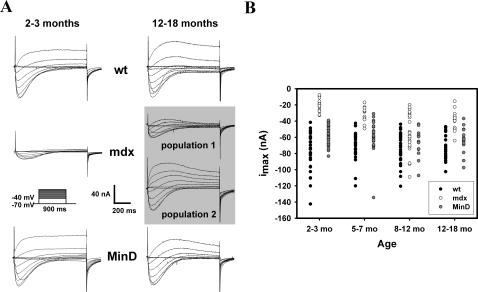
Age dependent L-type Ca^2+^ current distributions in intact single wt, mdx and MinD fibres. A, Representative i_Ca_ recordings in young (2–3 months) and aged (12–18 months) single wt, mdx and MinD fibres. In the 2–3 months old mdx fibre, the current amplitudes are markedly reduced. In aged mdx mice, a second fibre type appears with i_Ca_ amplitudes similar to wt and MinD fibres. B, Age dependent distribution of i_max_ amplitudes for age-matched wt, mdx and MinD fibres. In younger mdx fibres, i_max_ values were considerably smaller than in wt or MinD fibres of age-matched littermates. In older mdx fibres, some fibres (‘wt-like’ mdx fibres) displayed i_max_ values similar to those found in wt or MinD fibres while the rest (‘mdx-like’ mdx fibres) still had small i_max_ values similar to younger mdx fibres.

Representative recordings of i_Ca_ in single wt, mdx and MinD fibres from 2–3 months old (left panel) and 12–18 months old mice (right panel) are shown in Fig. 3 A. For all ages, i_Ca_ amplitudes and kinetics were similar in fibres from wt and MinD animals. In contrast, i_Ca_ amplitudes were always markedly reduced in younger mdx animals. In the age group of 12–18 months, however, some fibres with i_Ca_ amplitudes similar to those in wt and MinD fibres can be seen. This is also illustrated in Fig. 3 B, showing the distribution of maximum peak i_Ca_ amplitudes (i_max_) from recordings in at least twelve individual wt, mdx and MinD fibres from matching age groups. For mdx mice, i_max_ values obtained from younger fibres (2–3 and 5–7 months) are consistently smaller compared to wt and MinD fibres of the same age. However, in older mdx mice (8–12 and 12–18 months), several fibres with much larger values, similar to those found in wt and MinD fibres, as well as a larger population of age mdx fibres with i_max_ values similar to those found in the younger mdx age groups can be seen. The distribution of i_max_ values for wt and MinD fibres was stable with age. Interestingly, we detected no correlation between fibre morphology and i_max_ distribution in the older mdx fibres, as branched or split fibres were found in similar proportions with either reduced ‘mdx-like’ i_max_ or ‘wt-like’ i_max_ values (data not shown). To elucidate whether these different i_max_ values were also reflected by i_Ca_ activation kinetics, time-to-peak (TTP) was analyzed in each mdx subpopulation (‘mdx-like’ vs ‘wt-like’ mdx fibres). TTP was not significantly different (P>0.19) in the fibres with small ‘mdx-like’ i_max_ values for 8–12 months old fibres (178±48 ms at −10 mV, 138±17 ms at 0 mV and 112±11 ms at +10 mV) compared to those fibres with ‘wt-like’ larger i_max_ values (TTP: 160±21 ms, 116±8 ms and 103±8 ms, respectively). For 12–18 months old mdx fibres, there was also no significant difference for all potentials.

**Figure 4:**
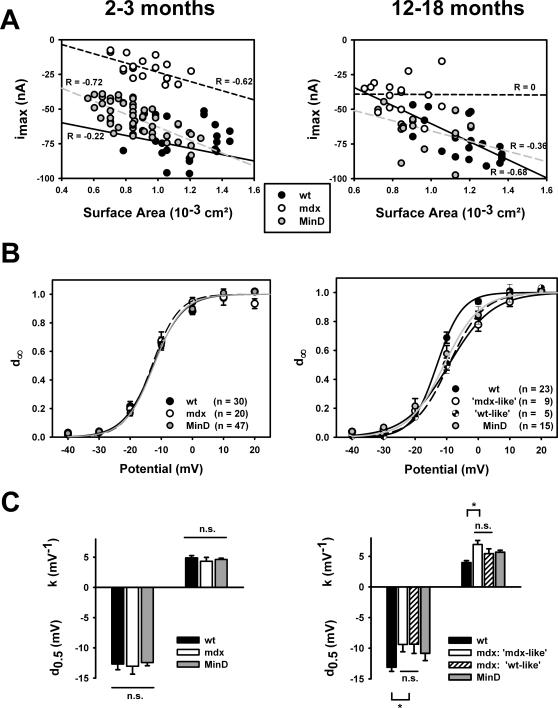
Dependence of i_Ca_ amplitudes on fibre dimensions and steady-state activation in single fibres from wt, mdx and MinD mice at 2–3 months and 12–18 months of age. A, i_max_ from individual fibres plotted against fibre surface area A revealed a linear relationship. In young fibres (2–3 months, left), the slope was comparable for the three genotypes and did not change in old wt and MinD fibres but was almost flat in mdx fibres (12–18 months, right). B, Activation curves reconstructed from the mean activation d_∞_ of all individual fibres of each strain, as given in the Methods. There is a perfect match in young fibres that is also reflected in the mean half-activation potentials d_0.5_ and slope factors k (C). In fibres from 12–18 months old mice, mdx fibres had significantly right-shifted activation curves. However, when looking at those mdx fibres with overlapping i_max_ values to wt fibres (see Fig. 3 B), these ‘wt-like’ mdx fibres had no difference in their activation curves compared to the ‘mdx-like’ mdx fibres with small i_max_ values (C). n.s.: not significant. *: P<0.05.

The i_max_ values in Fig. 3 Β are given as raw amplitudes. Previously, we showed that membrane capacitances were similar in the three strains [25]. i_max_ amplitudes, however, might still be skewed by differences in fibre sizes, as indeed many mdx fibres were smaller than wt fibres [25]. On the other hand, some mdx fibres may be bigger when compared to wt fibres [47]. It is, therefore, of interest to have a closer look at the relation of i_max_ to membrane surface area A as a measure for fibre size. Fig. 4 A shows the i_max_ values from all individual fibres of young (2–3 months) and aged (12–18 months) mice of the three strains plotted against their corresponding fibre surface A. There was a linear i_max_-A correlation that was comparable between the strains and showed an expected increase in i_max_ with increasing A in the young age group. In the older age group, this behaviour did not change for wt and MinD fibres, however, there was no more correlation for mdx fibres (R = 0) when considering the whole mdx fibre population. Interestingly, when one takes a closer look at the data, there are some fibres with increasing i_max_ upon increasing A, as well as fibres with decreasing i_max_.

In our previous study, we ruled out voltage-dependent inactivation as a source for the decreased i_max_ amplitudes in mdx fibres [25]. However, a possible shift in the steady-state activation curves of i_Ca_, i.e. a right-shift of d_∞_, might explain this reduction. We, therefore, calculated the d_∞_ curves for all individual fibres. Fig. 4 Β shows the mean d_∞_ values along with the mean reconstructed d_∞_ fit for all single fibres of the young (2–3 months) and older (12–18 months) age group in the three strains. As can be seen, there is a perfect match between the strains in the young age group, where the difference in i_max_ was most pronounced between mdx and wt or MinD fibres (Fig. 3 B). Half-activation potentials d_0.5_ and slope factors k were virtually identical in this age group (Fig. 4 C). This rules out a shift in d_∞_ as the cause for the reduced i_max_ amplitudes in mdx fibres. In the older age group, there was a right-shift in the d_∞_ curve of mdx fibres of ∼5 mV that could potentially explain some reduction of i_max_. However, when we compared the d_∞_ curves of the subgroup of mdx fibres that had overlapping i_max_ amplitudes with wt fibres (in Fig. 3 B) with those mdx fibres that had smaller amplitudes, there was virtually no difference in activation parameters (Fig. 4 C). We call the first subgroup ‘wt-like’ mdx fibres and the latter ‘mdx-like’ mdx fibres from their overlap with i_max_ values in the wt fibres. This analysis shows that, in the older age group, a shift in d_∞_ cannot account for the reduced i_max_ values in ‘mdx-like’ as compared to ‘wt-like’ mdx fibres, a finding that might have gone undetected when treating all mdx fibres alike in the analysis.

### Quantification of membrane dystrophin: different patterns in fibres from aged mdx animals

**Figure 5:**
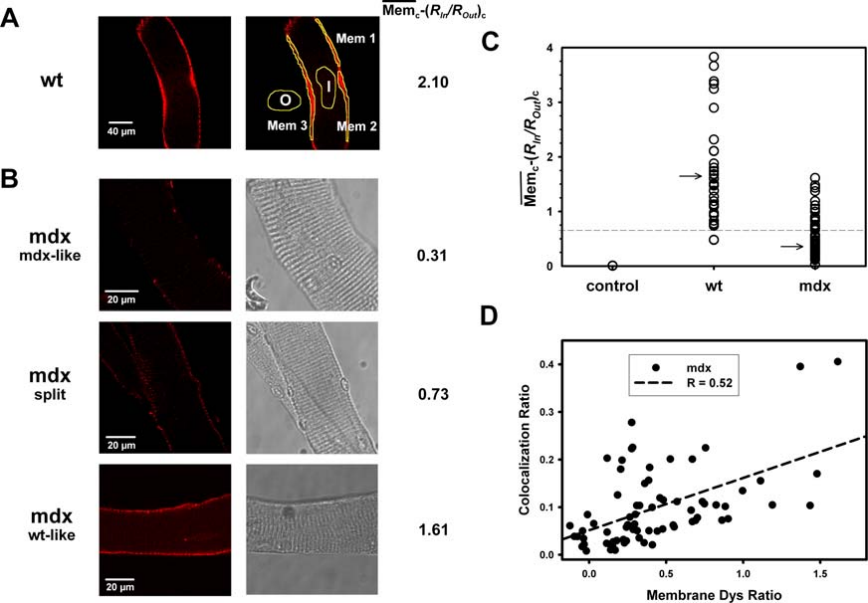
Quantification of membrane dystrophin signals identifies single ‘revertant’ mdx fibres. A, Membrane dystrophin signal intensities, manually selected from three ROIs, corrected for non-specific binding and related to the corrected signal from an intracellular ROI in a 10 months old wt fibre. The individual relative membrane signal 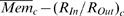 is also shown (wt: 2.10). B, ‘in situ’ dystrophin signals from three individual representative mdx fibres (10 months). From their morphological appearance, split fibres and fibres without apparent visual morphological abnormalities were classified. In the latter group, fibres were subdivided in ‘mdx-like’ (low 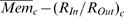 values) and ‘wt-like’ (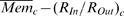 values comparable to wt fibres). C, the majority of mdx fibres from aged animals were ‘mdx-like’ and a small portion ‘wt-like’ (‘revertant’) as judged from the overlap of the wt lower quartile (dashed line) and the mdx upper quartile. Arrow: median. D, the degree of colocalization correlates with membrane Dys ratios 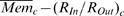 in aged mdx fibres.

We next investigated whether the functional phenotype diversity in the genetic mdx background was also reflected in the morphological data from the dystrophin immuno-fluorescence in living cells. For this, we quantified the dystrophin signals from membrane regions in images from 8–12 months old wt and mdx single fibres. For each image, the membrane signal was calculated from the average of three manually selected membrane ROIs (example shown in Fig. 5 A), as explained in the Methods. Fig. 5 B shows processed images from representative dystrophin labelled mdx fibres. Mdx fibres were grouped by morphology as either showing a dystrophic phenotype (branching and splitting, ‘split fibres’) or not. Fibres from the non-dystrophic phenotype group were further classified according to their dystrophin signal intensities as (i) ‘mdx-like’ with no or almost no membrane signal, or (ii) ‘wt-like’, i.e. having 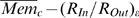 values comparable to those found in wt fibres (Fig. 5, A and C). The distribution of dystrophin signals from age-matched wt and mdx fibres is shown in Fig. 5 C. The control condition, with only secondary antibodies present, is properly predicted (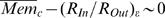), thus validating our approach. wt fibres showed a broad range of membrane dystrophin signals (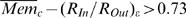, except for one fibre showing a value of 0.47). A few wt fibres had dystrophin signal values between 5 and 10 (not shown in Fig. 5 C). Signals from both wt and mdx fibres were not normally distributed and had values for the upper quartile of 3.97 (wt) vs. 0.68 (mdx), a median (arrow in Fig. 5 C) of 1.67 (wt) vs. 0.31 (mdx) and lower quartile of 1.16 (wt) vs. 0.16 (mdx). Thus, within the mdx distribution, most fibres showed much lower dystrophin signals than wt fibres. However, in the upper mdx quartile, some mdx fibres displayed dystrophin signals similar to those in the lower quartile of wt fibres and were, therefore, classified as ‘wt-like’. When focusing on the data from split mdx fibres, values ranged between 0.11 and 0.75. This suggests that split fibres are more ‘mdx-like’ and probably do not reflect ‘revertants’. On the other hand, when plotting 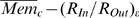 values against colocalization ratios (Fig. 5 D), their correlation (R = 0.52, P<0.001) suggest that ‘revertant’ fibres, in most cases, may restore the DHPR-dystrophin geometry.

## Discussion

### Live-cell confocal immuno-fluorescence of dystrophin and DHPR in single skeletal muscle fibres

The role of skeletal muscle L-type Ca^2+^ channels (Ca_v_1.1) in the pathophysiological mechanism of Duchenne muscular dystrophy has not been well characterized to date. This is particularly important when considering the time course of the disease, with initial phases of vast degeneration and ongoing degeneration/regeneration cycles, which can even take place in the same fibre simultaneously [55] and give rise to morphological abnormalities in aged fibres such as branching or splitting [10], [56]. An abnormal Ca^2+^ homeostasis has been suggested, but has also been experimentally controversial due to various experimental conditions and differentiation stages of the skeletal muscles used [12], [13], [16], [24], [29], [31], [51], [57]. Our previous study on L-type Ca^2+^ channel properties suggested that DHPR function was altered in mdx toe muscle fibres [25]. This implies that DHPR and dystrophin could interact either directly or indirectly. To further test this hypothesis, we aimed to quantify the degree of colocalization of DHPR with dystrophin in single isolated adult toe muscle fibres from wt, mdx and MinD mice. We developed a new ‘in situ’ confocal immuno-fluorescence technique in adult muscle fibres that involves a mild saponin permeabilization of relaxed single muscle fibres followed by antibody labelling. Imaging analysis involved a novel quantification algorithm that corrects for non-specific antibody binding, background signals and differences in overall staining intensities between fibres using appropriate normalization routines. To our knowledge, our study is the first to quantify dystrophin–ion channel colocalization in non-fixed adult muscle fibres. Previous approaches involving immuno-fluorescence in DMD have either relied on fibre fixation (e.g. [58]) or thin cryosections for immuno-histochemistry (e.g. [43], [44], [59]) and were of a qualitative nature. For example, the fixed cryosections given by Yokota et al. [59] show dystrophin-positive fibres at overview magnifications that contain about a dozen of fibres. Due to the slicing, only circumferences are stained and striation patterns cannot be resolved. In the mentioned study, fibres were classified as dystrophin-positive when more than 50% of the circumference was stained in a cross-section. Therefore, their binning was qualitatively binarized as ‘dystrophin-positive’ or ‘-negative’ [59], whereas we present a quantitative analysis of membrane dystrophin signals. A similar pattern was also observed in other cryosections [43]. Qualitatively, our immunofluorescence images are very similar compared to semi-thin cyrosections from mouse tibialis anterior muscle double-labelled for dystrophin and α-actinin (Fig. 3 in [60]). The authors also concluded that the dystrophin network was not uniformly distributed over the muscle fibre membrane in human, rat and mouse skeletal muscle [60]. Fixation procedures are known to disrupt or change the relative geometry of cellular compartments. For example, the relative volume of the t-tubules can dramatically change during fixation [61] or osmotic challenges, as compared to the resting condition [62]. The use of non-fixed, differentiated, single fibres in our study should reflect a more physiological environment of the DHPRs, which are almost exclusively located in the t-tubules in skeletal muscle [49]. Therefore, tubular colocalization quantifications of DHPR and dystrophin are expected to be more reliable in our unfixed preparations. The accuracy of the origin of our DHPR signal was independently tested using simultaneous two-photon imaging of DHPR and intrinsic SHG signals [54]. SHG and DHPR signal patterns were perfectly alternating (Fig. 1), as the SHG signal originates only from myosin with a maximum in the A-bands and two t-tubules per sarcomere located within the I-bands. The two I-bands originating from a Z-line into adjacent sarcomeres were not resolved in the multi-photon image shown but were present in images at higher magnification (not shown). It is important to note that we used laser settings to optimize the SHG signals over the DHPR signals when using the same excitation wavelength (880 nm) that might somewhat skew the resolution of the DHPR signals due to suboptimal quantum efficiency for the fluorescent dye. Therefore, only confocal single-photon images were used to quantify colocalization of DHPR and dystrophin.

### Colocalization of dystrophin and DHPR in wt, mdx and MinD fibres

Our data show a greater degree of DHPR-dystrophin colocalization in fibres from aged wt mice as compared to mdx fibres, with a median three times smaller for the latter. Colocalization was at 20–35% in most wt fibres. Interestingly, MinD fibres had a much larger median for colocalization. This implies a tighter coupling between the shorter mini-dystrophin and DHPR in transgenic MinD compared to wt fibres. Previous studies reported similar DHPR protein levels in wt, mdx [63] and MinD fibres [25], either ‘in vitro’ [63] or ‘in situ’ [25]. Therefore, it seems plausible that MinD, aside from its reconstitution to the subsarcolemmal membrane, could be expressed at higher levels in the tubules as compared to dystrophin in the wt. Our DHPR-dystrophin colocalization values in wt fibres provide a first quantitative analysis in adult skeletal muscle. In cardiac muscle, colocalization of DHPR and dystrophin has been qualitatively demonstrated in fixed cardiomyocytes from 4–8 wk old wt mice [44]. However, the overlap of both proteins had not been quantitatively analysed [44].

It was originally argued that dystrophin was only localized to the subsarcolemmal membrane [64], explaining its putative influence on sarcolemmal ion channels [57]. However, the localization of dystrophin has also been confirmed to the t-tubules of cardiac muscle [65] and the triads of skeletal muscle [66]. Moreover, an influence of dystrophin on DHPR function has recently been shown in electrophysiology studies of cardiac myocytes [44], [45], skeletal muscle mdx [67] and human DMD myotubes [68] and differentiated adult mdx fibres [51] over a wide age range [25].

Importantly, when looking closely at our colocalization data from mdx fibres (age: 10–12 months), one could already detect a small second population of mdx fibres with colocalization values similar to those found in wt fibres (Fig. 2 B).

### Two clusters of L-type Ca^2+^ currents in aged mdx fibres

So far, the morphological colocalization data suggest an increase in protein colocalization in some mdx fibres of middle-aged and older mice. If there was some functional direct/indirect coupling between DHPR and dystrophin, this should also be reflected in the L-type Ca^2+^ currents (i_Ca_). The most convincing argument for such a functional coupling comes from the i_Ca_ data in young mdx muscle fibres, where i_max_ values were consistently smaller compared to those found in wt and MinD fibres (Fig. 3 B and [25]). We previously ruled out voltage-dependent inactivation and activation/deactivation kinetics as an underlying cause for these i_max_ reductions [25]. In the present study, we were able to rule out a shift in steady-state activation d_∞_ as the cause for the reduced i_max_ values in young mdx fibres, as d_0.5_ and k values were perfectly matching (Fig. 4 B and C).

When carefully analyzing the i_Ca_ traces and i_max_ values from aged mdx mice, some fibres with i_max_ values almost identical to those found in wt and MinD fibres were found. The remaining mdx fibres still had small amplitudes, as seen in younger mdx fibres (Fig. 3 B). From their overlap of i_max_ values with wt fibres, we call the first population of mdx fibres ‘wt-like’ and the second ‘mdx-like’. The i_Ca_ activation and inactivation kinetics were the same for wt and mdx fibres of this age group regardless of amplitudes. Also, the activation curves of both ‘mdx-like’ and ‘wt-like’ mdx fibres were virtually the same although they both were slightly right-shifted about +5 mV compared to aged wt and MinD fibres (Fig. 4 B and C). Although this may explain some reduction of i_max_ values ‘per se’ in aged mdx fibres, it cannot explain the difference in amplitudes in the two subgroups.

When sub-analyzing the current kinetics and amplitudes with respect to morphological abnormalities, such as branching and splitting, we found no correlation between split fibres in older mdx mice and either only small or larger amplitudes in these fibres. This indicates that the degree of ongoing regeneration of skeletal muscle during aging of the mdx mice is not an indicator for restored DHPR function.

One could speculate that the raw values of i_max_, not normalized to capacitance or fibre surface in Fig. 3 B, might underestimate i_Ca_ amplitudes in smaller mdx fibres. Membrane capacitances in the three strains were previously reported to be similar [25]. Although a majority of mdx fibres was indeed smaller than wt or MinD fibres, there was still a linear dependence of i_max_ values with fibre surface A in young mdx fibres that was comparable to MinD or wt fibres (Fig. 4 A). Interestingly, in aged mdx fibres, the overall i_max_-A relation approached zero, indicating that fibre size and i_max_ values no longer correlated. Some larger mdx fibres had either very small or ‘wt-like’ large amplitudes and even very small fibres with large i_max_ values could be found in this age group (Fig. 4 A). This argues against a systematic underestimate in unnormalized i_max_ values in small mdx fibres. One can even say that ‘wt-like’ mdx fibres may be either small or large. The reason for this remains unclear.

In their study on voltage-clamped single fdb fibres from three to eight weeks old mdx animals, Collet et al. [51] did not observe reduced current amplitudes, which is in contrast to the findings in human myotubes [68] and mdx skeletal muscle (this study). However, the authors explained some of their scattering in i_Ca_ amplitudes to be a result of the possible existence of different subpopulations of mdx fibres as a consequence of distinct pathophysiological conditions [51]. In a recent patch-clamp study on mdx myotubes, Ca^2+^ channel potentiation was found to be reduced [67]. Finally, in mdx cardiac myocytes, absence of dystrophin shifted the steady-state activation of DHPR towards more positive potentials [44] or prolonged channel inactivation [45].

### Dystrophin-positive ‘revertant’ muscle fibres in aged mdx mice

It was hypothesized that during aging of the mdx mouse some fibre properties, which are impaired in the young animals, might be restored [19]. For example, from immuno-histochemistry, Hoffman et al. [41] found a significant correlation between the number of dystrophin-positive fibres that had undergone a spontaneous ‘reverse’ mutation in the mdx gene and the age of the mdx mouse [69]. Such fibres were also found in the diaphragm muscle of ten weeks old mdx4^cv^ mice [70]. To test whether membrane dystrophin signals in our mdx fibres were also clustered in a similar way to the i_Ca_ data, we quantified the amount of dystrophin signals originating from the membrane in all mdx fibres and compared them to wt fibres. The most robust parameter was the relative difference of sarcolemmal membrane signal and the signal from the fibre interior (both after correction for non-specific binding). This was markedly increased in wt fibres compared to the majority of mdx fibres. One can argue that this parameter would classify an mdx fibre that had both a strong membrane and internal dystrophin fluorescence signal as dystrophin-negative. However, this did not occur in images from mdx fibres (i.e. all fibres with small membrane-interior difference of the signal were faintly stained). Our results show an overlap of the lower quartile and the upper quartile for membrane dystrophin signals from wt and mdx fibres, respectively. This identifies dystrophin-positive ‘revertants’ as being at the lower range of ‘normal’ dystrophin expression in their wt counterparts. It has recently been described that the expansion of ‘revertants’ with age represents a cumulative history of regeneration in mdx fibres [59]. In that study, fibres were classified as ‘revertants’ when more than half of the fibre circumference was positively stained for dystrophin [59]. The regenerative re-expression of dystrophin seems to be accompanied by a restoration of its localization relative to DHPRs. This is confirmed by the colocalization-dystrophin signal relationship in our mdx fibres: mdx fibres with a ‘wt-like’ dystrophin pattern generally also had large ‘wt-like’ colocalization ratios. Interestingly, we did not confirm this relation when analyzing the ‘split fibre’ subgroup. This novel finding indicates that morphologically abnormal mdx fibres might not necessarily be ‘revertants’, albeit the successive degeneration/regeneration that must have occurred in these fibres. The reasons for this remain unclear.

One further interesting result from our ‘in-situ’ study in aged mdx fibres is that some ‘revertants’ seem to present a patchy membrane staining pattern for dystrophin rather than the continuous pattern found in wt fibres. This might have gone undetected in previous studies mainly involving immuno-histochemistry cryosections of mdx muscle [59]. It can be hypothesized that, during successive membrane damage in mdx fibres, the membrane scaffolds might be remodelled and, therefore, locally alter the dystrophin binding sites. Further research is needed to address this point.

With the applied techniques, we show that dystrophin and DHPR significantly colocalize in wt and MinD fibres, regardless of age. The same is true for middle-aged and older mdx fibres, mostly in cases where the almost ‘wt-like’ colocalization ratios correlate with a ‘wt-like’ membrane bound dystrophin pattern. The latter fibres can, therefore, be considered as ‘revertants’. These ‘revertants’ are most likely not predominantly those fibres that show morphological alterations, i.e. branching and splitting, as these generally showed ‘mdx-like’ dystrophin staining. The restored colocalization in ‘revertants’ also coincides with the restoration of ‘wt-like’ i_max_ amplitudes in a proportion of mdx fibres with age, suggesting a functional coupling. It cannot be ruled out that dystrophin exerts its effects on DHPR function in an indirect manner. The observed effects could be due to a coupled expression of a DHPR-regulating protein to dystrophin expression, or by the effects of a signalling cascade. The underlying molecular mechanism of this regulation will certainly have to be addressed in future studies.

### Implications for altered ec-coupling and Ca^2+^ homeostasis in DMD

**Figure 6:**
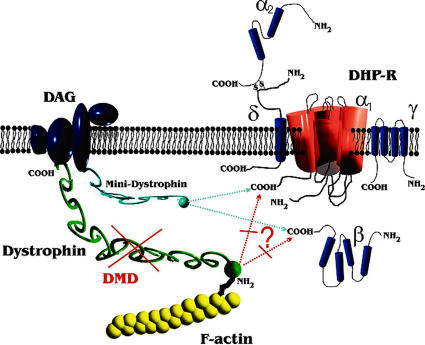
Proposed model of L-type Ca^2+^ channel (DHPR) interaction with dystrophin in skeletal muscle. Dystrophin normally binds to actin via its N-terminus. Some dystrophin molecules may bind directly/indirectly to the C-terminus of the α_1_-subunit or to the auxiliary ß-subunit, thereby modifying channel properties. In DMD, this linkage is suggested to be disrupted. In aged revertant mdx fibres, the putative link between the channel and the cytoskeleton is rescued (not shown). In MinD fibres, mini-dystrophin is suggested to functionally replace dystrophin. DAG: dystrophin-associated glycoprotein complex.

Lack of dystrophin has been shown to alter a variety of ion channels and pumps in muscle that, in most cases, are associated with intracellular Ca^2+^ handling [12], [13], [15], [24], [71]. Impaired Ca^2+^ homeostasis has been shown to result in chronic Ca^2+^ overload and muscle injury, in particular under conditions involving mechanical membrane stress, e.g. eccentric contractions [8], [9], [13]. In this scenario, it would be disadvantageous to have fully functional DHPR that can contribute to small Ca^2+^ influxes under conditions of tetanic contractions (as suggested in [35], [36]) or store-depletion [37]. Our current working hypothesis involves a model, in which dystrophin is linked either directly and/or indirectly to DHPRs, thus modifying their single channel properties (Fig. 6). Presumably, mechanical tension exerted on L-type Ca^2+^ channels by dystrophin and cytoskeleton components act to increase either single channel conductance, open probability or a combination of both. In young and aged ‘mdx-like’ mdx fibres, this would result in a reduction of i_Ca_. With regard to impaired Ca^2+^ homeostasis and potential Ca^2+^ overload in mdx fibres [72], [73], the absent dystrophin link to DHPR may, at least partly, compensate for increased Ca^2+^ influx through other pathways (e.g. store-operated channels, leak channels etc.) in young fibres from mdx mice, where degeneration predominates. This may help to limit the increase-or even stabilize-resting Ca^2+^ levels [25]. With ongoing regeneration, revertant fibres become apparent, and with the re-expression of dystrophin, the link might be restored and pathological Ca^2+^ influx pathways repaired. With a reduction in Ca^2+^ ‘leakage’ influx, DHPRs can become fully restored. It would be an interesting objective for future studies to investigate Ca^2+^ influx, e.g. from leak channels, in aged ‘revertants’. Additionally, the altered DHPR properties in mdx fibres might, in turn, reduce the coupling efficiency to the SR release channels and may be the missing link for the physiologically relevant impairment in the ec-coupling process, i.e. the impaired SR calcium release during action potentials in mdx mouse muscle fibres [74]. Collet et al. [51] found no differences in charge movements in single muscle fibres from 3–8 weeks old wt and mdx mice under their experimental conditions [51]. More research is needed to address the age dependence of charge movements in the three strains presented here in a larger sampling bin.

Lastly, one might expect a milder reduction of i_Ca_ amplitudes in mdx muscle fibres, as a large fraction of DHPR should not be directly affected by the absence of dystrophin because (i) dystrophin is poorly expressed in the t-tubules as compared to DHPR and (ii) colocalization in wt fibres was about one third. Our finding of an overall two third reduction of i_Ca_ in young mdx fibres, therefore, favours some additional indirect interaction of both constituents. For example, activation of phosphorylation pathways by protein kinase A was able to restore voltage-dependent potentiation of L-type Ca^2+^ channels in mdx fibres [67]. One also has to bear in mind that probably only a fraction of DHPR represents functional Ca^2+^ channels [75] and this could also determine the degree of regulation by dystrophin.

Our results show that mini-dystrophin is also a very potential regulator in skeletal muscle fibres. Our approach, therefore, offers additional tools to evaluate the functional restoration of skeletal muscle DHPR-dystrophin interaction in models involving adeno-associated virus transfection gene therapy using micro-dystrophins [43], [76] or exon-skipping [4].

## References

[1] Boland BJ, Silbert PL, Groover RV, Wollan PC, Silverstein MD (1996). Skeletal, cardiac and smooth muscle failure in Duchenne muscular dystrophy.. Pediatr Neurol.

[2] Melacini P, Vianello A, Villanova C, Fanin M, Miorin M (1996). Cardiac and respiratory involvement in advanced stage Duchenne muscular dystrophy.. Neuromuscul Disord.

[3] Chamberlain JS (2002). Gene therapy of muscular dystrophy.. Hum Mol Genetics.

[4] Denti MA, Rosa A, D'Antona G, Sthandier O, De Angelis FG (2006). Body-wide gene therapy of Duchenne muscular dystrophy in the mdx mouse model.. Proc Natl Acad Sci USA.

[5] Hoffman EP, Brown RH, Kunkel LM (1987). Dystrophin: the protein product of the Duchenne muscular dystrophy locus.. Cell.

[6] Blake DJ, Weir A, Newey SE, Davies KE (2002). Function and genetics of dystrophin and dystrophin-related proteins in muscle.. Physiol Rev.

[7] Ervasti JM, Campbell KP (1993). A role for the dystrophin-glycoprotein complex as a transmembrane linker between laminin and actin.. J Cell Biol.

[8] Moens P, Baatsen PHWW, Marechal G (1993). Increased susceptibility of EDL muscles from mdx mice to damage induced by contractions with stretch.. J Muscle Res Cell Motil.

[9] Petrof BJ, Shrager JB, Stedman HH, Kelly AM, Sweeney HL (1993). Dystrophin protects the sarcolemma from stresses developed during muscle contraction.. Proc Natl Acad Sci USA.

[10] Head SI, Williams DA, Stephenson DG (1992). Abnormalities in structure and function of limb skeletal muscle fibres of dystrophic mdx mice.. Proc R. Soc Lond B Biol Sci.

[11] Constantin B, Sebille S, Cognard C (2006). New insights in the regulation of calcium transfers by muscle dystrophin-based cytoskeleton: implications in DMD.. J Muscle Res Cell Motil.

[12] Divet A, Lompre AM, Huchet-Cadiou C (2005). Effect of cyclopiazonic acid, an inhibitor of the sarcoplasmic reticulum Ca-ATPase, on skeletal muscles from normal and mdx mice.. Acta Physiol Scand.

[13] Yeung EW, Whitehead NP, Suchyna TM, Gottlieb PA, Sachs F (2005). Effects of stretch-activated channel blockers on [Ca^2+^]_i_ and muscle damage in the mdx mouse.. J Physiol.

[14] Wang X, Weisleder N, Collet C, Zhou J, Chu Y (2005). Uncontrolled calcium sparks act as a dystrophic signal for mammalian muscle.. Nat Cell Biol.

[15] Woods CE, Novo D, DiFranco M, Capote J, Vergara JL (2005). Propagation in the transverse tubular system and voltage dependence of calcium release in normal and mdx mouse muscle fibres.. J Physiol.

[16] Tutdibi O, Brinkmeier H, Rüdel R, Fohr KJ (1999). Increased calcium entry into dystrophin-deficient muscle fibres of mdx and adr-mdx mice is reduced by ion channel blockers.. J Physiol.

[17] Vandebrouck A, Ducret T, Basset O, Sebille S, Raymond G (2006). Regulation of store-operated calcium entries and mitochondrial uptake by minidystrophin expression in cultured myotubes.. FASEB J.

[18] Coirault C, Lambert F, Pourny JC, Lecarpentier Y (2002). Velocity of actomyosin sliding in vitro is reduced in dystrophic mouse diaphragm.. Am J Respir Crit Care Med.

[19] Fink RHA, Stephenson DG, Williams DA (1990). Physiological properties of skinned fibres from normal and dystrophic (Duchenne) human muscle activated by Ca^2+^ and Sr^2+^.. J Physiol (London).

[20] Messina S, Altavilla D, Aguennouz M, Seminara P, Minutoli L (2006). Lipid peroxidation blunts nuclear factor κB activation, reduces skeletal muscle degeneration, and enhances muscle function in mdx mice.. Am J Pathol.

[21] Messina S, Bitto A, Aguennouz M, Minutoli L, Monici MC (2006). Nuclear factor κB blockade reduces skeletal muscle degeneration and enhances muscle function in mdx mice.. Exp Neurol.

[22] Chakkalakal JV, Michel SA, Chin ER, Jasmin BJ (2006). Targeted inhibition of Ca^2+^/calmodulin signalling exacerbates the dystrophic phenotype in mdx mouse muscle.. Hum Mol Genet.

[23] Senter L, Ceoldo S, Petrusa MM, Salviati G (1995). Phosphorylation of dystrophin: effects on actin binding.. Biochem Biophys Res Commun.

[24] Kumar AK, Khandelwal N, Malya R, Reid MB, Boriek AM (2004). Loss of dystrophin causes aberrant mechanotransduction in skeletal muscle fibers.. FASEB J.

[25] Friedrich O, Both M, Gillis JM, Chamberlain JS, Fink RHA (2004). Mini-dystrophin restores L-type calcium currents in skeletal muscle of transgenic mdx mice.. J Physiol.

[26] Turner PR, Fong PY, Denetclaw WF, Steinhardt RA (1991). Increased calcium influx in dystrophic muscle.. J Cell Biol.

[27] Fong PY, Turner PR, Denetclaw WF, Steinhardt RA (1990). Increased activity of calcium leak channels in myotubes of Duchenne human and mdx mouse origin.. Science.

[28] De Backer F, Vandebrouck C, Gailly P, Gillis JM (2002). Long-term study of Ca^2+^ homeostasis and of survival in collagenase-isolated muscle fibres from normal and mdx mice.. J Physiol.

[29] Gailly P, Boland B, Himpens B, Casteels R, Gillis JM (1993). Critical evaluation of cytosolic calcium determination in resting muscle fibres from normal and dystrophic (mdx) mice.. Cell Calcium.

[30] Allard B, Couchoux H, Pouvreau S, Jacquemond V (2006). Sarcoplasmic reticulum Ca^2+^ release and depletion fail to affect sarcolemmal ion channel activity in mouse skeletal muscle.. J Physiol.

[31] Vandebrouck A, Martin D, Colson-Van Shoor M, Debaix H, Gailly P (2002). Involvement of TRPC in the abnormal calcium influx observed in dystrophic mdx mouse skeletal muscle fibres.. J Cell Biol.

[32] Weisleder N, Brotto M, Komazaki S, Pan Z, Zhao X (2006). Muscle aging is associated with compromised Ca^2+^ spark signalling and segregated intracellular Ca^2+^ release.. J Cell Biol.

[33] Wang ZM, Messi ML, Delbono O (2000). L-type Ca^2+^ channel charge movement and intracellular Ca^2+^ in skeletal muscle fibres from aging mice.. Biophys J.

[34] Ge Y, Molloy MP, Chamberlain JS, Andrews PC (2004). Differential expression of the skeletal muscle proteome in mdx mice at different ages.. Electrophoresis.

[35] Francini F, Bencini C, Squecco R (1996). Activation of L-type calcium channel in twitch skeletal muscle fibres of the frog.. J Physiol.

[36] Melzer W, Herrmann-Frank A, Lüttgau HC (1995). The role of Ca^2+^ ions in excitation-contraction coupling of skeletal muscle fibres.. Biochim Biophys Acta.

[37] Kurebayashi N, Ogawa Y (2001). Depletion of Ca^2+^ in the sarcoplasmic reticulum stimulates Ca^2+^ entry into mouse skeletal muscle fibres.. J Physiol.

[38] Gonzalez-Serratos H, Valle-Aguilera R, Lathrop DA, del Carmen Garcia M (1982). Slow inward calcium currents have no obvious role in muscle excitation-contraction coupling.. Nature.

[39] Lyfenko AD, Goonasekera SA, Dirksen RT (2004). Dynamic alterations in myoplasmic Ca^2+^ in malignant hyperthermia and central core disease.. Biochem Biophys Res Commun.

[40] MacLennon DH (2000). Ca^2+^ signalling and muscle disease.. Eur J Biochem.

[41] Hoffman EP, Morgan JE, Watkins SC, Partridge TA (1990). Somatic reversion/suppression of the mouse mdx phenotype in vivo.. J Neur Sci.

[42] Phelps S, Hauser MA, Cole NM, Rafael JA, Hinkle RT (1995). Expression of full-length and truncated dystrophin mini-genes in transgenic mdx mice.. Hum Mol Genet.

[43] Gregorevic P, Blankinship MJ, Allen JM, Crawford RW, Meuse L (2004). Systemic delivery of genes to striated muscles using adeno-associated viral vectors.. Nat Med.

[44] Sadeghi A, Doyle AD, Johnson BD (2002). Regulation of the cardiac L-type Ca^2+^ channel by the actin-binding protein alpha-actinin and dystrophin.. Am J Physiol Cell Physiol.

[45] Woolf PJ, Lu S, Cornford-Nairn RA, Watson M, Xia XH (2006). Alterations in dihydropyridine receptors in dystrophin-deficient cardiac muscle.. Am J Physiol Heart Circ Physiol.

[46] Bulfield G, Siller WG, Wight PAL, Moore KJ (1984). X-chromosome-linked muscular dystrophy (mdx) in the mouse.. Proc Natl Acad Sci USA.

[47] Harper SQ, Hauser MA, DelloRusso C, Duan D, Crawford RW (2002). Modular flexibility of dystrophin: implications for gene therapy of Duchenne muscular dystrophy.. Nat Med.

[48] Friedrich O, Ehmer T, Fink RHA (1999). Calcium currents during contraction and shortening in enzymatically isolated murine skeletal muscle fibres.. J Physiol.

[49] Almers W, Fink R, Palade PT (1981). Calcium depletion in frog muscle tubules: the decline of calcium current under maintained depolarization.. J Physiol.

[50] Friedrich O, Ehmer T, Uttenweiler D, Vogel M, Barry PH (2001). Numerical analysis of Ca^2+^ depletion in the transverse tubular system of mammalian muscle.. Biophys J.

[51] Collet C, Csernoch L, Jacquemond V (2003). Intramembrane charge movement and L-type calcium current in skeletal muscle fibres isolated from control and mdx mice.. Biophys J.

[52] Bangham AD, Horne RW, Glauert AM, Dingle JT, Lucy JA (1962). Action of saponin on biological cell membranes.. Nature.

[53] Launikonis BS, Stephenson DG (1997). Effect of saponin treatment on the sarcoplasmic reticulum of rat, cane toad and crustacean (yabby) skeletal muscle.. J Physiol.

[54] Both M, Vogel M, Friedrich O, v Wegner F, Künsting T (2004). Second harmonic imaging of intrinsic signals in muscle fibres in situ.. J Biomed Opt.

[55] Pastoret C, Sebille A (1995). Age-related differences in regeneration of dystrophic (mdx) and normal muscle in the mouse.. Muscle Nerve.

[56] Pastoret C, Sebille A (1993). Mdx mice show progressive weakness and muscle deterioration with age.. J Neurol Sci.

[57] Allard B (2006). Sarcolemmal ion channels in dystrophin-deficient skeletal muscle fibres.. J Muscle Res Cell Motil.

[58] Marchand E, Constantin B, Balghi H, Claudepierre MC, Cantereau A (2004). Improvement of calcium handling and changes in calcium-release properties after mini- or full-length dystrophin forced expression in cultured skeletal myotubes.. Exp Cell Res.

[59] Yokota T, Lu QL, Morgan JE, Davies KE, Fisher R (2006). Expansion of revertant fibres in dystrophic mdx muscles reflects activity of muscle precursor cells as an index of muscle regeneration.. J Cell Sci.

[60] Straub V, Bittner RE, Leger JJ, Voit T (1992). Direct visualization of the dystrophin network on skeletal muscle fiber membrane.. J Cell Biol.

[61] Dulhunty A (1984). Heterogeneity of t-tubule geometry in vertebrate skeletal muscle fibres.. J Muscle Res Cell Motil.

[62] Launikonis BS, Stephenson DG (2002). Tubular system volume changes in twitch fibres from toad and rat skeletal muscle assessed by confocal microscopy.. J Physiol.

[63] Pereon Y, Dettbarn C, Navarro J, Noireaud J, Palade PT (1997). Dihydropyridine receptor gene expression in skeletal muscle from mdx and control mice.. Biochim Biophys Acta.

[64] Cullen MJ, Walsh J, Nicholson LVB, Harris JB (1990). Ultrastructural localization of dystrophin in human muscle by using gold immuno-labelling.. Proc R. Soc Lond (Biol).

[65] Frank JS, Mottino G, Chen F, Peri V, Holland P (1994). Subcellular distribution of dystrophin in isolated adult and neonatal cardiac myocytes.. Am J Physiol Cell Physiol.

[66] Hoffman EP, Knudson CM, Campbell KP, Kunkel LM (1987). Subcellular fractionation of dystrophin to the triads of skeletal muscle.. Nature.

[67] Johnson BD, Scheuer T, Catterall W (2005). Convergent regulation of skeletal muscle Ca^2+^ channels by dystrophin, the actin cytoskeleton, and cAMP-dependent protein kinase.. Proc Nat Acad Sci USA.

[68] Imbert N, Vandebrouck C, Duport G, Raymond G, Hassoni AA (2001). Calcium currents and transients in co-cultured contracting normal and Duchenne muscular dystrophy human myotubes.. J Physiol.

[69] Matsumura K, Tome FMS, Collin H, Leturcq F, Jean-Pierre M (1994). Expression of dystrophin-associated proteins in dystrophin-positive muscle fibers (revertants) in Duchenne muscular dystrophy.. Neuromuscul Disord.

[70] Decrouy A, Renaud JM, Davis HL, Lunde JA, Dickson G (1997). Mini-dystrophin gene transfer in mdx4cv diaphragm muscle fibers increases sarcolemmal stability.. Gene Ther.

[71] McCarter GC, Steinhardt RA (2000). Increased activity of calcium leak channels caused by proteolysis near sarcolemmal ruptures.. J Membr Biol.

[72] Alderton JM, Steinhardt RA (2000). Calcium influx through calcium leak channels is responsible for the elevated levels of calcium-dependent proteolysis in dystrophic myotubes.. J Biol Chem.

[73] Mallouk N, Allard B (2002). Ca^2+^ influx and opening of Ca^2+^-activated K^+^ channels in muscle fibers from control and mdx mice.. Biophys J.

[74] Woods CE, Novo D, DiFranco M, Vergara JL (2004). The action potential-evoked sarcoplasmic reticulum calcium release is impaired in mdx mouse muscle fibres.. J Physiol.

[75] Schwartz LM, McCleskey EW, Almers W (1985). Dihydropyridine receptors in muscle are voltage-dependent but most are not functional calcium channels.. Nature.

[76] Liu M, Yue Y, Harper SQ, Grange RW, Chamberlain JS (2005). Adeno-associated virus-mediated microdystrophin expression protects young mdx muscle from contraction-induced injury.. Mol Ther.

